# Viscoelastic parameters derived from multifrequency MR elastography for depicting hepatic fibrosis and inflammation in chronic viral hepatitis

**DOI:** 10.1186/s13244-024-01652-5

**Published:** 2024-03-26

**Authors:** Yikun Wang, Jiahao Zhou, Huimin Lin, Huafeng Wang, Ingolf Sack, Jing Guo, Fuhua Yan, Ruokun Li

**Affiliations:** 1 Department of Radiology, Ruijin Hospital, Shanghai Jiao Tong University School of Medicine, No. 197 Ruijin Er Road, 200025 Shanghai, China; 2 Department of Phathology, Ruijin Hospital, Shanghai Jiao Tong University School of Medicine, No. 197 Ruijin Er Road, 200025 Shanghai, China; 3 Department of Radiology, Berlin Institute of Health, Charité-Universitätsmedizin Berlin, Corporate Member of Freie Universität Berlin, Humboldt-Universität zu Berlin, Berlin, Germany; 4 College of Health Science and Technology, Shanghai Jiao Tong University School of Medicine, Shanghai, China

**Keywords:** Fibrosis, Inflammation, Liver, Magnetic resonance elastography

## Abstract

**Objectives:**

The capability of MR elastography (MRE) to differentiate fibrosis and inflammation, and to provide precise diagnoses is crucial, whereas the coexistence of fibrosis and inflammation may obscure the diagnostic accuracy.

**Methods:**

In this retrospective study, from June 2020 to December 2022, chronic viral hepatitis patients who underwent multifrequency MRE (mMRE) were included in, and further divided into, training and validation cohorts. The hepatic viscoelastic parameters [shear wave speed (*c*) and loss angle (*φ*) of the complex shear modulus] were obtained from mMRE. The logistic regression and receiver operating characteristic (ROC) curves were generated to evaluate performance of viscoelastic parameters for fibrosis and inflammation.

**Results:**

A total of 233 patients were assigned to training cohort and validation cohorts (mean age, 52 years ± 13 (SD); 51 women; training cohort, *n* = 170 (73%), and validation cohort, *n* = 63 (27%)). Liver *c* exhibited superior performance in detecting fibrosis with ROC (95% confidence interval) of ≥ S1 (0.96 (0.92–0.99)), ≥ S2 (0.86 (0.78–0.92)), ≥ S3 (0.89 (0.84–0.95)), and S4 (0.88 (0.83–0.93)). Similarly, *φ* was effective in diagnosing inflammation with ROC values of ≥ G2 (0.72 (0.63–0.81)), ≥ G3 (0.88 (0.83–0.94)), and G4 (0.92 (0.87–0.98)). And great predictive discrimination for fibrosis and inflammation were shown in validation cohort (all AUCs > 0.75).

**Conclusion:**

The viscoelastic parameters derived from multifrequency MRE could realize simultaneous detection of hepatic fibrosis and inflammation.

**Critical relevance statement:**

Fibrosis and inflammation coexist in chronic liver disease which obscures the diagnostic performance of MR elastography, whereas the viscoelastic parameters derived from multifrequency MR elastography could realize simultaneous detection of hepatic fibrosis and inflammation.

**Key points:**

• Hepatic biomechanical parameters derived from multifrequency MR elastography could effectively detect fibrosis and inflammation.

• Liver stiffness is useful for detecting fibrosis independent of inflammatory activity.

• Fibrosis could affect the diagnostic efficacy of liver viscosity in inflammation, especially in early-grade of inflammation.

**Graphical Abstract:**

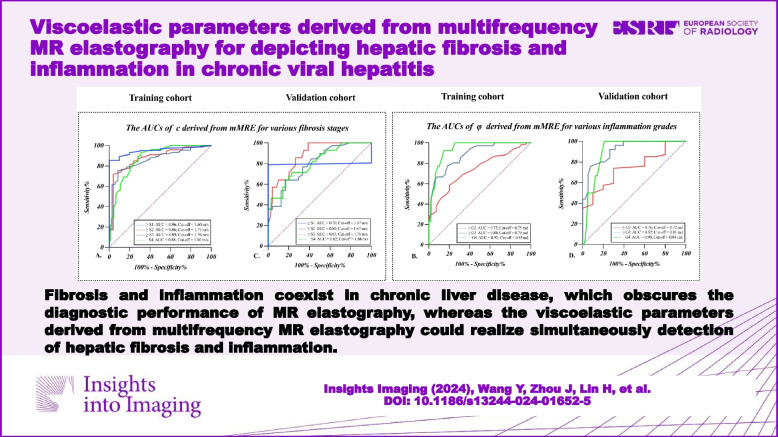

**Supplementary Information:**

The online version contains supplementary material available at 10.1186/s13244-024-01652-5.

## Introduction

Chronic liver diseases caused by viral hepatitis infections represent a significant global health concern [1, 2]. Fibrosis, a well-documented consequence of these diseases, is a dynamic process that can be reversed with appropriate treatment, particularly in its early stages [3]. Hence, timely identification of fibrosis and inflammation is of paramount importance for determining clinical management and improving prognosis [4]. Although liver biopsy has been the worldwide reference standard for staging fibrosis and inflammation, it has some limitations such as invasiveness, potential complications, and sampling error due to heterogeneity [5, 6]. Consequently, there is an urgent need to develop non-invasive and low-risk methods for staging hepatic fibrosis and inflammation.

Previous research has indicated that MR elastography (MRE) is as accurate as liver biopsy and offers excellent performance in this regard [7–9]. However, earlier studies have noted that changes in liver shear stiffness (SS) derived from two-dimensional (2D) MRE can be challenging to distinguish between inflammatory activity and the degree of fibrosis [8, 10]. Therefore, there is a necessity for multiparametric three-dimensional (3D) MR elastography, which reflects more mechanical properties of the tissue. A recent publication by Yu Shi et al. demonstrated that 3D MRE, utilizing a single frequency (60 Hz), effectively stages fibrosis and inflammation while distinguishing necroinflammation from liver fibrosis using liver shear stiffness (SS) and damping ratio (DR) [11].

Similar to 3D MRE, the multifrequency MR elastography (mMRE) employs a multifrequency approach in the range of 30 to 60 Hz to measure hepatic viscoelastic parameters. Previous study has demonstrated that the diagnostic performance of single-frequency (45 Hz, 55 Hz, 60 Hz) and compound multifrequency processing is equivalent for staging hepatic fibrosis with shear wave speed (c) [12]. However, the additional value derived from mapping the phase angle (*φ*) of the complex shear modulus (also known as the loss angle) remains to be fully explored [13].

Although previous results reported that higher inflammation activity was associated with higher liver stiffness by increasing local blood availability, inflammatory cell infiltration, and interstitial pressure [9, 14, 15], the intricate connection between fibrosis and inflammation may obscure the diagnostic accuracy. Therefore, the objective of this retrospective study is to assess the diagnostic utility of viscoelastic parameters derived from mMRE in characterizing hepatic fibrosis and inflammation in viral hepatitis patients and further explore the intrinsic of fibrosis and inflammation by viscoelastic parameters.

## Materials and methods

This retrospective study was approved by the institutional review board of Ruijin Hospital and the research ethics committees of the participating centers. Furthermore, the informed consent requirement was waived for this retrospective study.

### Study population

**Fig. 1:**
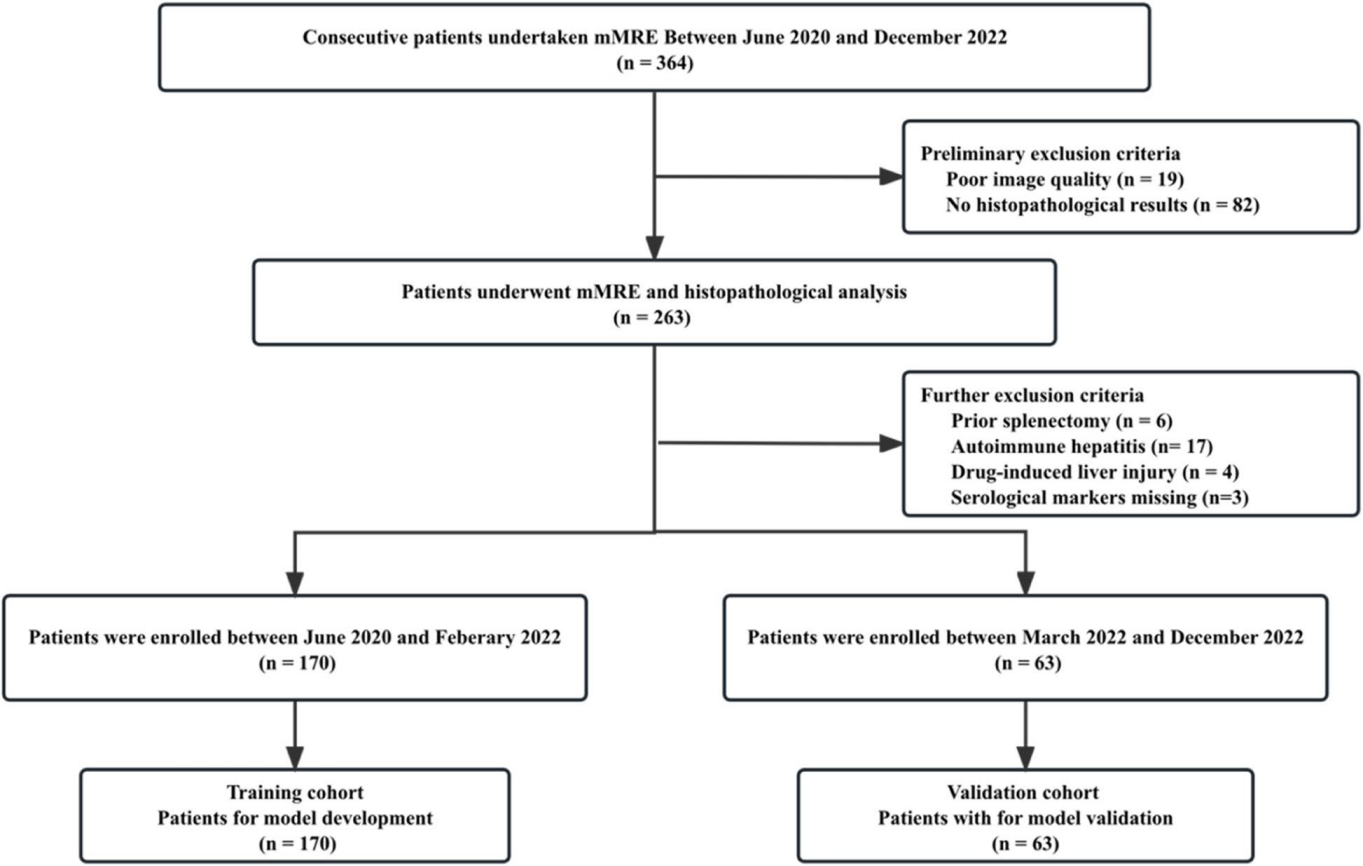
Flowchart of study population. *mMRE* = multifrequency magnetic resonance elastography, *CHB* chronic hepatitis B, *CHC* chronic hepatitis C

Between June 2020 and December 2022, 364 consecutive patients who underwent mMRE were included in this study. Histopathological analysis was conducted within 1 week following the mMRE examination. A total of 131 patients were further excluded due to technical failure of MRE (*n* = 19), absent histopathology results (*n* = 82), missing serological markers (*n* = 3), prior splenectomy (*n* = 6), and/or diagnosis of non-hepatic B and C (*n* = 21). Finally, 233 chronic viral B and C hepatitis patients were ultimately included and were divided into the training cohort (between June 2020 and February 2022, *n* = 170) and validation cohort (between March 2022 and December 2022, *n* = 63) according to the time period in which their scans were obtained [16]. The schematic of patient selection is provided as Fig. 1. The basic clinical characteristics were retrospectively collected from the hospital information system.

### MRI and MRE acquisition

MRI sequences were performed at a 1.5-T scanner (Magnetom Aera, Siemens, Erlangen, Germany). All patients were fasted for a minimum of 4–6 h prior to examination.

All participants underwent routine multiparametric MRI, which consisted of T1- and T2-weighted imaging sequences (Supplementary Table 1). MRE sequence was also performed at a 1.5-T scanner. Two posterior (0.4 bar) and two pneumatic actuators (0.6 bar) were placed near the liver and spleen region. The three-dimensional wave fields were acquired using a single-shot, spin-echo echo planar imaging (SE-EPI) planar MRI sequence with flow-compensated motion-coding gradients (MEGs).

Fifteen contiguous transverse slices were obtained during free breathing. The imaging parameters of sequence were as follows: repetition time of 15.40 ms; echo time of 2.40, 4.80, 7.10, 9.50, 11.90, and 14.30 ms; field of view of 312 × 384 mm^2^; slice thickness 5 mm; flip angle 5°; a matrix size of 104 × 128, and a resolution of 3 × 3 × 5 mm^3^. Additional imaging parameters were detailed as follows: MEG frequencies were set at 43.48 Hz for vibration frequencies of 30, 40, and 50 Hz, and 44.88 Hz for a vibration frequency of 60 Hz; MEG amplitude was set at 30 mT/m; repetition time was 2050 ms; echo time was 59 ms. The total acquisition time for the complete set of mMRE data was approximately 3.5 min. Additional details about the MRE sequence are presented in Supplementary Table 2.

### Image analysis

The mMRE data were processed using the processing pipeline available at https://bioqic-apps.com [17]. Full field-of-view high-spatial resolution maps of shear wave speed (*c*) and loss angle of the complex shear modulus (*φ*) were generated using a multifrequency wave number-based processing algorithm (k-MDEV) and Laplacian operators-based processing method (MDEV) [13]. The *c* (m/s) and *φ* (rad) were referred to as surrogates of stiffness and viscosity, respectively.

Two radiologists independently and manually drew regions of interest (ROIs) by contouring livers while excluding large blood vessels and bile ducts based on the magnitude images respectively [18]. The average of the measurement on three consecutive slices represents the *c* and *φ*. Two data sets were used to evaluating the reproducibility of measurements.

### Histopathological analysis

The hepatic pathologic specimens were obtained by liver resection and percutaneous liver biopsy, which were carried out for histopathological analysis with hematoxylin-eosin (HE) staining. A pathologist (with 15 years of experience in liver pathology) conducted the analysis while remaining blinded to radiological and clinical results. The fibrosis stage and inflammation grade were determined according to the Scheuer system. The degree of liver tissue damage was expressed by inflammation grade (grading, G) and fibrosis stage (staging, S). Grading was based on the degree of necrosis and inflammation, assessing the activity of the disease; staging was based on the degree of fibrosis and formation of cirrhosis, indicating the progress of the disease [19, 20]. Fibrosis staging was categorized as follows: mild fibrosis (S ≥ 1), significant fibrosis (S ≥ 2), advanced fibrosis (S ≥ 3), and cirrhosis (S = 4). Inflammation grading included mild (G ≥ 2), moderate (G ≥ 3), and severe inflammatory activity (G = 4), with active necroinflammation defined as G ≥ 2. Additional details about the Scheuer system are presented in Supplementary Table 3.

### Statistical analysis

Differences in characteristics among the training, validation data were assessed using Student’s *t* test and Fisher exact. The interobserver agreement of measurements was evaluated by the intraclass correlation coefficient (ICC) and Bland-Altman analysis. The Kruskal-Wallis was applied to evaluate the differences of viscoelastic parameters under various stages of fibrosis and grades of inflammatory activity (*p* values were adjusted by the Bonferroni correction). The univariable and multivariable logistic regression analysis were tested to assess the independent factors of fibrosis and inflammation, and further receiver operating characteristic (ROC) curves were used to analyze the diagnostic efficacy of the parameters, and area under the ROC curves (AUROCs) was calculated with 95% confidence intervals (CIs), and the optimal cutoff value was determined with the Youden index. The concordance index was applied to estimate the efficacy of viscoelastic parameters.

All statistical analyses were performed by SPSS (version 26.0; SPSS, Chicago, III) and GraphPad Prism (version 9.0, GraphPad). A *p-*value < 0.05 was considered indicative of statistically significant difference.

## Results

### Patients’ characteristics

Two hundred thirty-three chronic viral B and C hepatitis patients were ultimately included, with 170 patients (74%) in the training cohort, and 63 (26%) in the validation cohort. The baseline demographic and clinical characteristics of patients are summarized in Table 1. Compared with validation cohort, the training cohort had a lower proportion of women (*p* = 0.04), lower alanine transaminase (*p* = 0.005), and total bilirubin (*p* = 0.009).

**Table 1:** The baseline characteristics of patients in training and validation cohort

Characteristic	Training cohort	Validation cohort	*p* value
General	170^a^	63^a^	
Gender	135:35^a^	47:16^a^	0.04*
Age(years)	56 (54–58)	57 (54–60)	0.69
Biochemical data			
BMI(kg/m^2^)	23.92 (23.56–24.47)	23.64 (22.95–24.34)	0.75
PLT(×10^9^/L)	149.54 (137.64–161.40)	142.49 (127.82–157.35)	0.19
ALT(IU/L)	51.29 (38.58–63.95)	72.01 (54.75–89.29)	0.005*
AST(IU/L)	48.82 (36.92–60.72)	59.19 (26.89–91.49)	0.27
TB(µmol/L)	20.33 (17.61–23.01)	28.53 (21.88–35.19)	0.009*
DB(µmol/L)	5.66 (3.65–7.67)	8.26 (4.41–12.09)	0.09
APRI	1.36 (0.88–1.83)	1.03 (0.86–1.20)	0.68
FIB-4	3.65 (2.91–4.39)	3.94 (1.48–6.40)	0.59
Histopathology			
Fibrosis stage			
S0	10^a^	1^a^	
S1	23^a^	11^a^	
S2	37^a^	12^a^	
S3	18^a^	11^a^	
S4	82^a^	28^a^	
Inflammatory activity			
G0	7^a^	1^a^	
G1	45^a^	9^a^	
G2	82^a^	28^a^	
G3	23^a^	19^a^	
G4	13^a^	6^a^	

Except where indicated, data are means, with 95% CIs in parentheses

*BMI* Body mass index, *PLT* Platelet count, *ALT* Alanine transaminase, *AST* Aspartate transaminase, *TB* Total bilirubin, *DB* Direct bilirubin, *APRI* Aspartate transaminase-to-platelet ratio index score, *FIB-4* Fibrosis-4 index

^*^Statistically significant difference between training and validation cohort (*p* < 0.05)

^a^Data are numbers of patients

### Interobserver variability in measuring of *c* and *φ* values

**Fig. 2:**
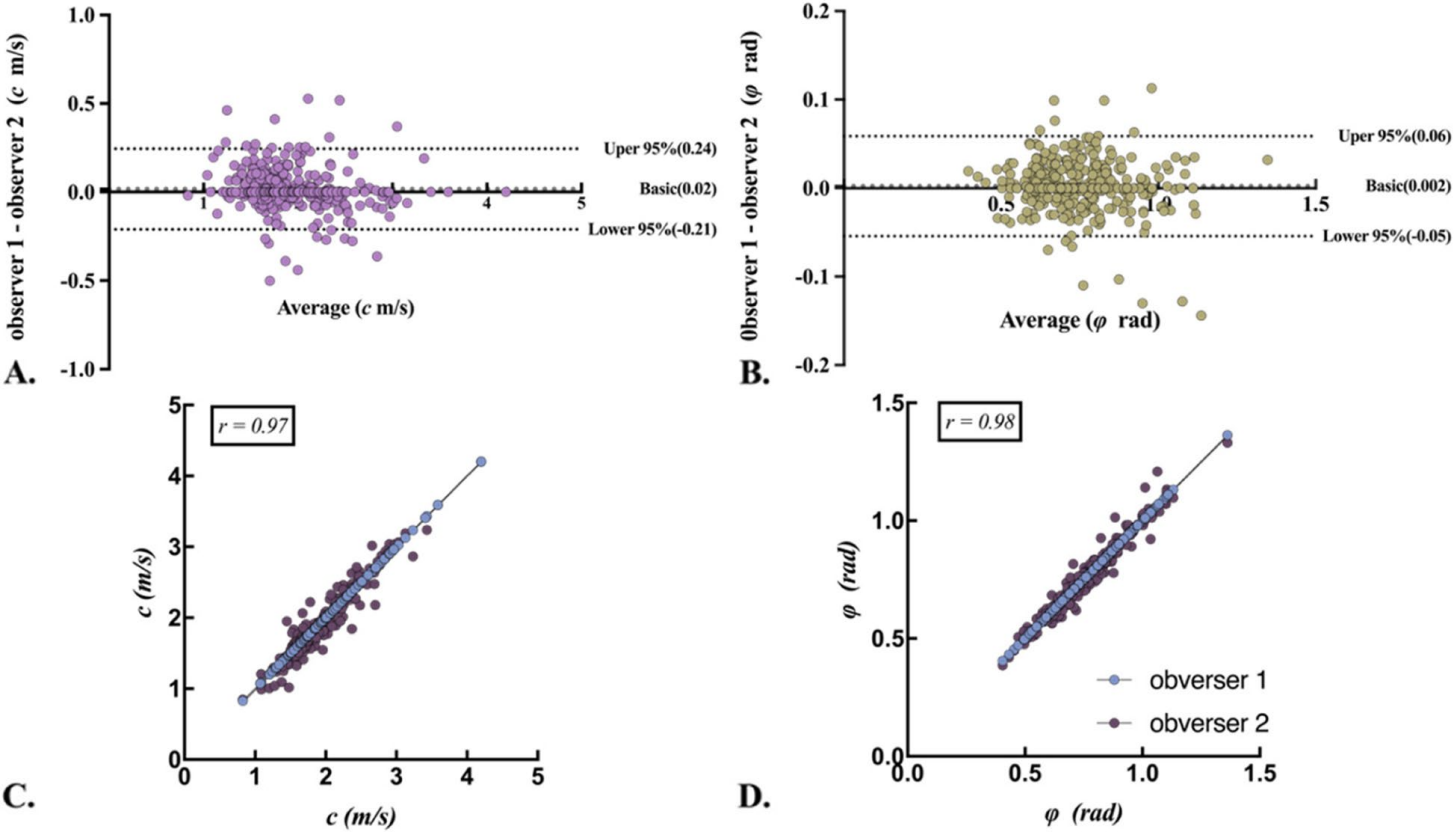
The Bland-Altman plots of the measured shear wave speed (*c*) and loss angle of the complex shear modulus (*φ*) of observer 1, 2. The* c* and *φ* values demonstrate good internal consistency reliability (ICC = 0.97 and 0.98, *p* < 0.001), respectively

As illustrated in Fig. 2, there was excellent consistency of* c* and* φ* between the two observers, with intraclass correlation coefficients (ICC) of (0.97 (95% CI 0.96–0.97)) for c and (0.98 (95% CI 0.97–0.98)) for *φ*, respectively.

### The distribution and regression of hepatic viscoelastic parameters in fibrosis and inflammation

**Fig. 3:**
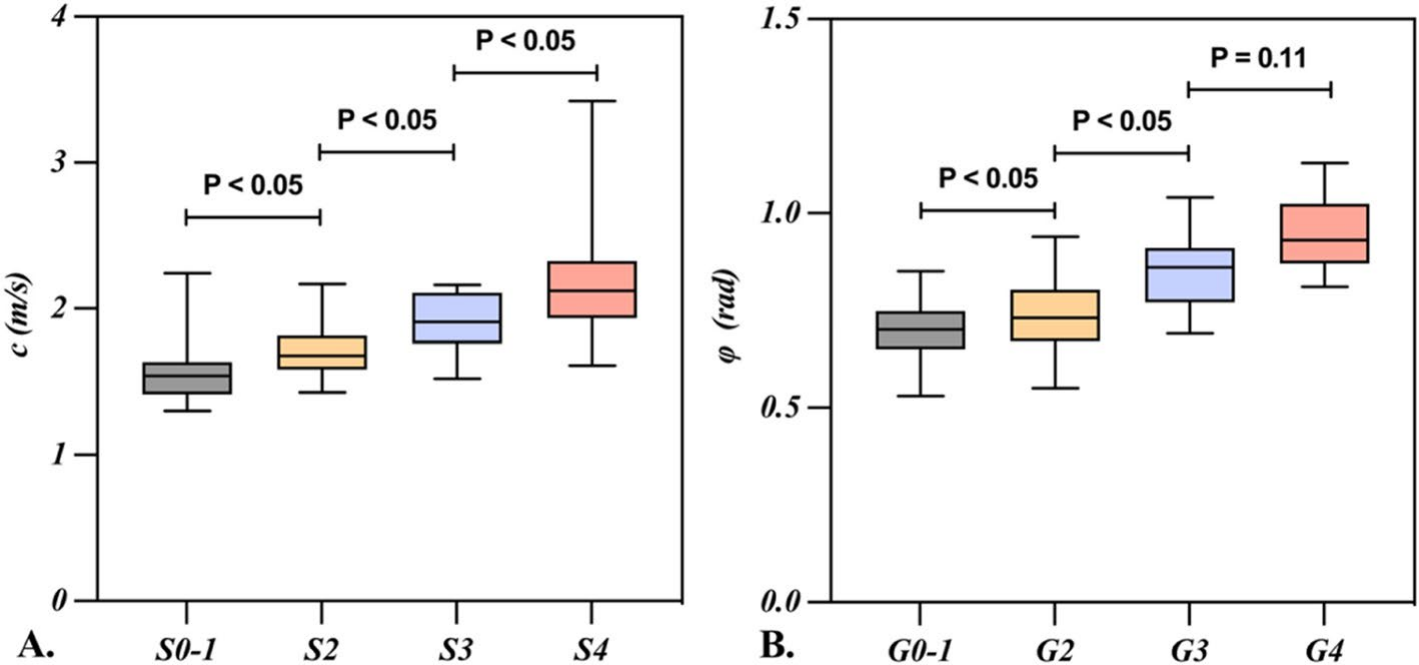
The boxplots represent the distribution of *c* and* φ* in patients with chronic hepatitis. Median, upper, and lower quartile and whiskers of *c*,* φ* are displayed. The lower and upper borders correspond to the first and third quartiles (the maximum and minimum value). The midline indicates the median. As shown in **A**, there was significant difference in the *c* among the patients at each fibrosis stage. **B** As for *φ*, there was also significant difference at each inflammation grade except for the comparison between G3 and G4

As presented in Fig. 3 and Supplementary Table 4, *c* value significantly increased with the progression of fibrosis. Similarly, *φ* value also demonstrated the similar tendency except for the comparison between G3 and G4 (*p* = 0.11). Further details are provided in Table 2 and Supplementary Table 4. Additionally, both univariable and multivariate logistic regression analyses indicated that *c* was an independent indicator for fibrosis, while *φ* for inflammation (Supplementary Table 5).

**Table 2:** Distribution of stages of fibrosis and grades of inflammation in training cohort

Fibrosis stage	Grade of inflammatory activity				Total
	G0–1	G2	G3	G4	
S0	8	2	0	0	10
S1	15	7	0	1	23
S2	10	22	4	1	37
S3	4	9	4	1	18
S4	15	42	15	10	82
Total	52	82	23	13	170

*S* Staging, fibrosis stage; *G* Grading, inflammation grade

**Fig. 4:**
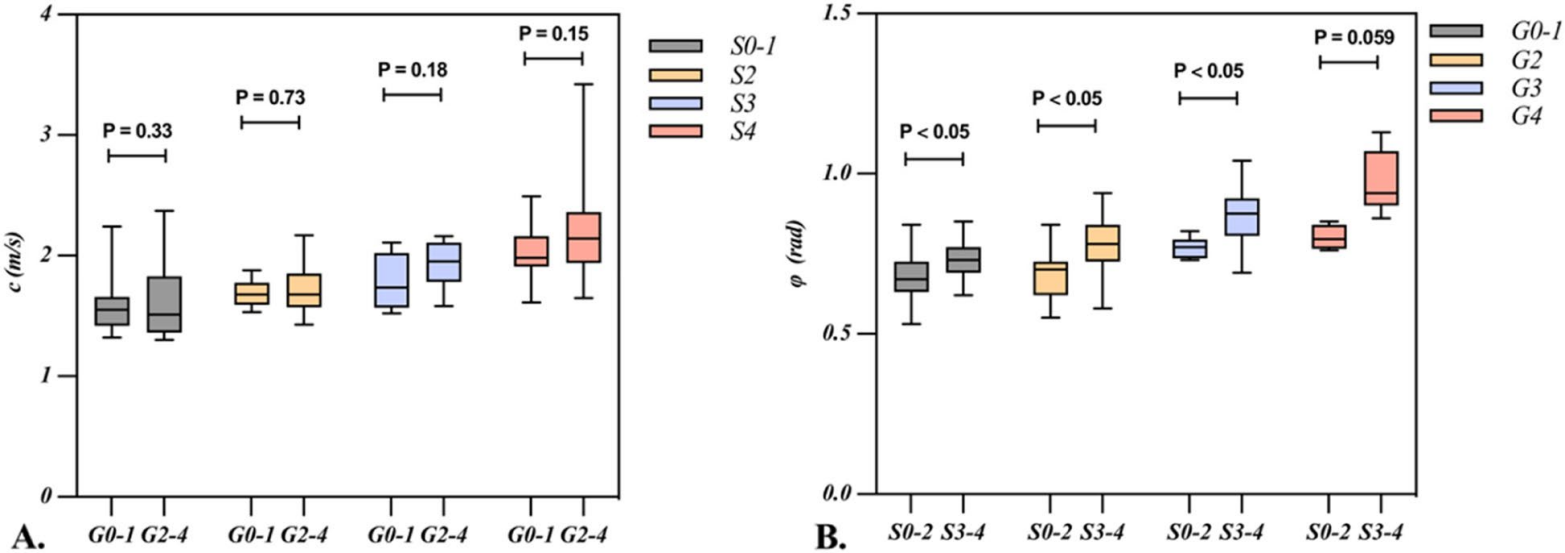
Scatter plots represent measurement of *c* and* φ*, assessed with mMRE, in patients with different fibrosis and inflammation stages. The lower and upper borders correspond to the first and third quartiles (the maximum and minimum value). The Boxplots (**A**) illustrated that no significant difference in *c* values at match fibrosis stages was found between no active inflammation (G0-1) group and active inflammation (G2-4) group (*p* > 0.05). The boxplots (**B**) illustrate there were significant difference in *φ* values at each inflammation stage between no advanced fibrosis (S0-3) group and advanced fibrosis (S3-4) group (G4, borderline statistical significance, *p* = 0.059)

Subgroup analysis, aimed at investigating the interaction between fibrosis and inflammation, revealed that active necro-inflammation (G ≥ 2) had no significant impact on* c* at each fibrosis stage (all *p* > 0.05). In addition, at each inflammation grade, advanced fibrosis (S ≥ 3) had significantly positive impact on *φ* ([S0-2 vs. S3-4] G0-1, 0.68 ± 0.08 vs 0.73 ± 0.06; G2, 0.68 ± 0.08 vs 0.77 ± 0.08; and G3, 0.77 ± 0.04 vs 0.87 ± 0.09; all *p* < 0.05) except for severe inflammatory activity (G4) with borderline statistical significance (*p* = 0.059) (Fig. 4). These results collectively suggest that *c* might be influenced by fibrosis, while* φ* was influenced by the interaction with fibrosis and inflammation.

### Diagnosis performance of viscoelastic parameters for fibrosis and inflammation

**Fig. 5:**
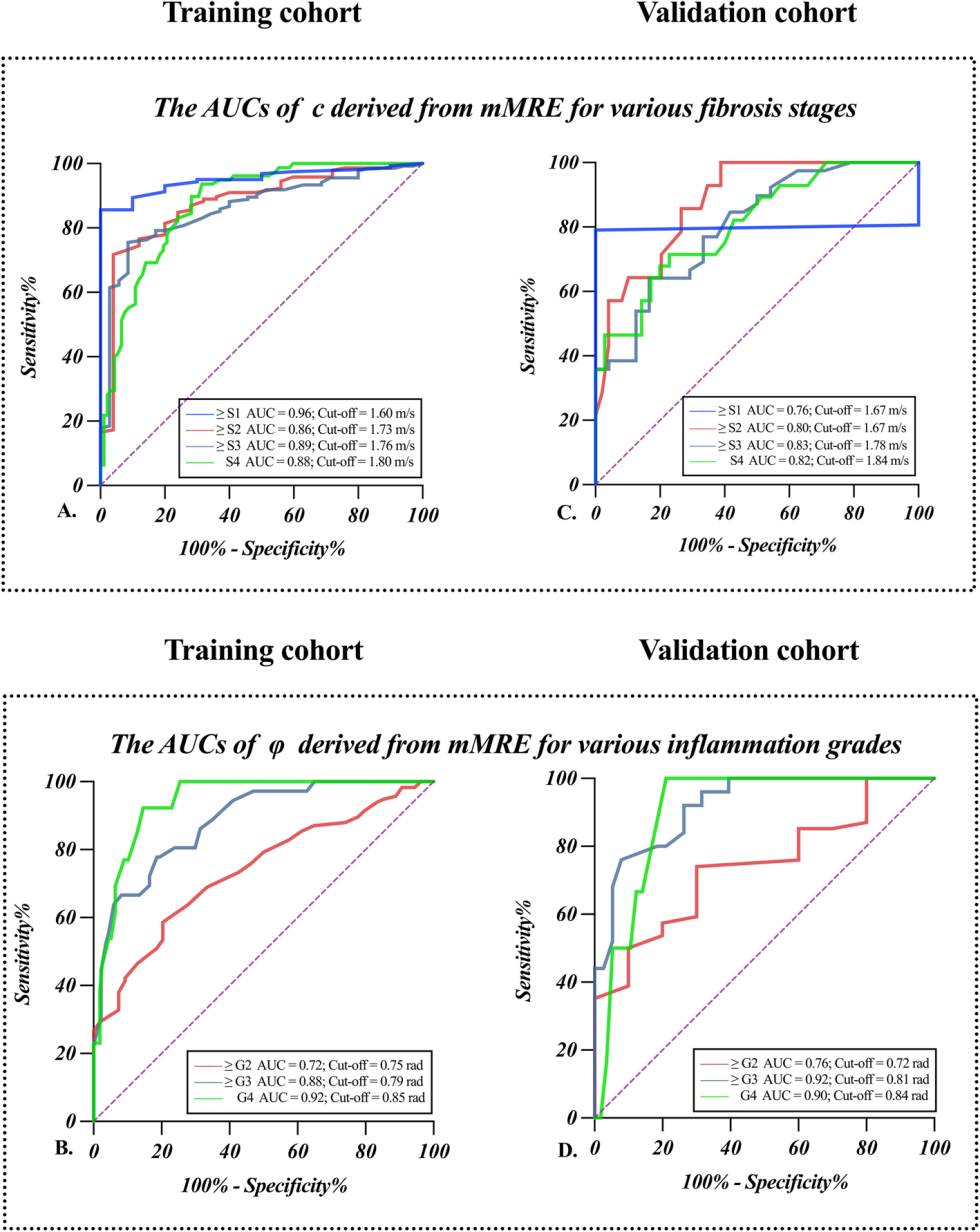
Graphs show area under the receiver operating characteristic curves (AUC) for viscoelastic parameters derived mMRE for diagnosing fibrosis in (**A**) training cohort, and (**C**) validation cohort, as well as inflammation (**B**) in training cohort, and (**D**) in validation. There were superior diagnostic performances in training cohort for various fibrosis stages (AUC: from 0.86 to 0.96, *p* < 0.05) and inflammatory activity grades (AUC: from 0.72 to 0.92, *p* < 0.05). The viscoelastic parameters were fair to good with high discriminatory capability for diagnosing fibrosis and inflammation (all AUCs > 0.75)

As presented in Table 3 and Fig. 5, the parameter *c* exhibited superior diagnostic performance for detecting mild fibrosis (≥ S1), significant fibrosis (≥ S2), advanced fibrosis (≥ S3), and cirrhosis (S4), with respective AUC values of 0.96 (95%CI, 0.92–0.99), 0.86 (95% CI, 0.78–0.92), 0.89 (95% CI, 0.84–0.95), and 0.88 (95% CI, 0.83–0.93). Regarding inflammation, parameter *φ* also showed good diagnostic capability for grading mild inflammation (≥ G2), moderate inflammation (≥ G3), and severe inflammation (G4), with AUCs of 0.72 (95% CI, 0.63–0.81), 0.89 (95% CI, 0.83–0.94), and 0.92 (95% CI, 0.87–0.98), respectively.

**Table 3:** Diagnostic performance in staging various fibrosis and grading various inflammatory activity in training and validation cohorts

	Cut-off	AUC (95%CI)	Sensitivity (%)	Specificity (%)		Cut-off	AUC (95%CI)	Sensitivity (%)	Specificity (%)
Training cohort									
*c*					*φ*				
≥ S1	1.60 m/s	0.96 (0.92–0.99)	85.6	100	≥ G2	0.75 rad	0.72 (0.63–0.81)	60.2	76.9
≥ S2	1.73 m/s	0.86 (0.78–0.92)	77.8	75.7	≥ G3	0.79 rad	0.88 (0.83–0.94)	81.1	76.7
≥ S3	1.76 m/s	0.89 (0.84–0.95)	91.8	78.1	G4	0.85 rad	0.92 (0.87–0.98)	85.7	85.9
S4	1.80 m/s	0.88 (0.83–0.93)	92.3	72.8					
Validation cohort									
*c*					*φ*				
≥ S1	1.67 m/s	0.76 (0.65–0.93)	100	62.3	≥ G2	0.72 rad	0.76 (0.61–0.90)	75.5	70.0
≥ S2	1.67 m/s	0.80 (0.79–0.97)	73.5	85.7	≥ G3	0.81 rad	0.92 (0.85–0.98)	76.0	92.1
≥ S3	1.78 m/s	0.83 (0.70–0.92)	64.2	83.3	G4	0.84 rad	0.90 (0.82–0.98)	100	80.7
S4	1.84 m/s	0.82 (0.72–0.92)	71.4	77.1					

*rad* Radian, *CI* Confidence interval, *c* Shear wave speed, *φ* Loss angle of the complex shear modulus, *S* Staging, fibrosis stage, *G* Grading, inflammation grade

In the validation cohort, *c* demonstrated fair to good discriminatory ability in depicting fibrosis with AUCs from 0.76 to 0.83. Similarly, *φ* exhibited excellent discriminatory capability for grading inflammation with AUCs from 0.76 to 0.92. The details are described in Fig. 5 and Table 3.

## Discussion

In this study, we evaluated the diagnostic performance of hepatic viscoelastic parameters (shear wave speed [*c*] and loss angle of the complex shear modulus [*φ*]) derived from mMRE for characterizing fibrosis and inflammation respectively. Additionally, we investigated the interplay between fibrosis and inflammation. Our findings revealed that *c* emerged as an independent predictor for staging fibrosis, while *φ* proved valuable for grading inflammation. The area under the ROC curves for staging fibrosis with* c* were (0.86–0.96) in training cohort, and (0.76 to 0.83) in validation cohort. Similarly, excellent performances were observed for grading various inflammation with *φ* in all cohorts (training cohort 0.72–0.92 and validation cohort 0.76 to 0.92).

Our study introduced parameters *c* and *φ* to quantify the stiffness and viscosity of liver tissue. In our research, we found *c* to be a robust indicator for diagnosing fibrosis, exhibiting high sensitivity and specificity, although it failed to detect inflammation. And further subgroup analysis indicated that active necroinflammation (≥ G2) did not significantly influence *c* at matched fibrosis stages, meaning that stiffness might be barely influenced by active necroinflammation, contrasting with previous results that reported that higher inflammation activity was significantly associated with higher stiffness [9, 11, 14, 15, 21]. While previous studies have indicated that hepatic inflammation may stimulate hepatic stellate cells (HSCs), leading to increased collagen production and deposition in interstitial collagen fibrils and the extracellular matrix (ECM) [22, 23], it is essential to note that the hepatic histological lesions associated with inflammation are typically linked to hepatocyte necrosis and apoptosis, including periportal and intralobular necrosis [20, 24]. It has been established that tissue necrosis could result in a decrease in the measurement of tissue stiffness [25]. Consequently, the variability in liver stiffness is influenced by differences in the extent of collagen deposition and tissue necrosis, which could be a reasonable explanation for above difference.

Interestingly, as an independent risk factor for detecting inflammation, *φ* performed well in grading inflammation, though there was significant difference at *φ* values between advanced and non-advanced fibrosis groups (expect for G4 with marginal statistical significance, *p* = 0.059). As a result, we deduced that *φ* could serve as an alternative indicator for inflammation, whereas it was not only influenced by inflammation but also fibrosis.

While our findings partially contrasted with previous study on 3D MRE characterizing fibrosis and inflammation, which demonstrated the capability of hepatic viscosity to detect early necroinflammation (prior to fibrosis) largely influenced by advanced fibrosis [11], the capability for detecting moderate and severe inflammation was notably superior. We speculate that an uneven distribution of fibrosis and inflammation may be one of the factors. More than half of patients with mild inflammation (29/52, 56%) have advanced fibrosis, which may influence diagnostic accuracy. This also explains the results observed in our study where viscosity values exhibited relatively limited diagnostic efficacy in early inflammation. In addition, the diagnostic performance of viscosity may be influenced by the variations in liver function parameters and the prevalence of underlying chronic liver diseases, with chronic hepatitis B being predominant in our study.

To summarize, taking into consideration the coexistence of liver fibrosis and inflammation in patients with chronic liver disease, utilizing multiple biomechanical parameters based on mMRE, which reflect the degree of liver fibrosis and inflammatory activity, can make a more accurate assessment of the degree of fibrosis and inflammatory activity. Liver stiffness exhibited high sensitivity and specificity in detecting various stages of fibrosis, making it a reliable non-invasive diagnostic marker which could alert clinicians to the presence of liver fibrosis in patients, and reduce the need for liver biopsy when possible [26]. Although liver viscosity may not exhibit high sensitivity in early-stage inflammation, its significant specificity makes it a valuable exclusionary diagnostic tool. However, the varying diagnostic performance of viscosity may be significantly influenced by this dual dependence on both liver fibrosis and inflammation, as well as the diverse characteristics of different populations in terms of these factors. Therefore, given the rich diagnostic information provided by both stiffness and viscosity, clinicians should consider the specific clinical context of each patient when assessing the progression of chronic liver disease based on mMRE results.

Our study also has some limitations that should be considered when interpreting findings. First, despite the relatively large cohort study of patients, the distribution of fibrosis and inflammation stages particularly G4 was uneven. Second, as a retrospective study, there may have been selection bias, whereby most of patients had an underlying disease of chronic hepatitis B. Third, liver fibrosis and inflammation fibrosis were evaluated using liver biopsy or histopathology as the reference standard, which may have led to interobserver and interregional variability. The relatively small sample size of histopathology specimens compared with the large liver volumes assessed by mMRE also may cause mismatch results. Finally, this is a single-center retrospective study, and the diagnostic efficacy of viscosity value may still be affected by different population characteristics and potential confounding factors, which still needs to be validated at the multicenter level.

In conclusion, building upon previous research, this study contributes to better understanding of the intrinsic development of fibrosis and inflammation in chronic liver disease. The results of this study demonstrated that mMRE could effectively and simultaneously detect hepatic fibrosis and inflammation. Furthermore, it underscores that fibrosis could affect the diagnostic efficacy of viscosity in inflammation, especially in early-grade of inflammation. In light of these results, we further recommend utilizing the stiffness parameter (*c*) for diagnosing fibrosis and the viscosity parameter (*φ*) for assessing inflammation. Therefore, multifrequency MRE, known for its practicality and reliability, has promising clinical applications.

### Supplementary Information

**Supplementary Material 1.**

## Data Availability

The research data involves personal privacy, and if necessary, contact the corresponding author to obtain it.

## Abbreviations

3D: Three-dimensional
AUC: Area under the receiver operating characteristic curve
CI: Confidence interval
mMRE: Multifrequency magnetic resonance elastography
