# Supplementary material for: Viscoelastic parameters derived from multifrequency MR elastography for depicting hepatic fibrosis and inflammation in chronic viral hepatitis
Source: Insights Imaging. 2024 Mar 26;15:91. doi: 10.1186/s13244-024-01652-5 (PMC10965837; doi:10.1186/s13244-024-01652-5)
Supplement: Supplementary file 1 — Supplementary Material 1. [file 13244_2024_1652_MOESM1_ESM.pdf]

Viscoelastic parameters derived from multifrequency MR elastography for depicting hepatic fibrosis and inflammation in chronic viral hepatitis
ELECTRONIC SUPPLEMENTARY MATERIAL

Supplementary Table 1: Multiparametric MRI protocol

	T1-weighted	T2- weighted	
	Axial	Axial	Coronal
Repetition time (msec)	4.85	3000	1200
Echo time (msec)	2.41	95	91
Flip angle (degree)	10	140	180
Field of view (mm^2)	380×309	380×380	380×380
Matrix size	320×240	320×320	256×256
Section thickness (mm)	3	5	6

Supplementary Table 2: Pulse Sequence parameters for multifrequency MR elastography

Parameters	Multifrequency MR elastography Aera (SIEMENS)
Filed Strength (T)	1.5
Sequence	SE-EPI
Echo time (msec)	59.0
Repetition time (msec)	2050
Field of view (mm^2)	312×384
Matrix	128×104
Resolution (mm^2)	3× 5 × 3
Number of Slices	15
Slice Thickness (mm)	4
Slice Gap (mm)	2
Frequency (Hz)	30, 40, 50, 60
Encoding Direction	x, y, z
Breath Control	Free breathing
Imaging time (min)	3:30

Note: SE-EPI = spin-echo echo planar imaging

Supplementary Table 3: The Scheuer scoring System for necro-inflammatory activity and fibrosis in Chronic Hepatitis

A simple system for Scoring necro-inflammatory activity, fibrosis and cirrhosis in Chronic Hepatitis		
Grade	Portal/Periportal Activity	Lobular Activity
0	None or minimal	None
1	Portal inflammation	Inflammation but no necrosis
2	Mild piecemeal necrosis	Focal necrosis or acidophil bodies
3	Moderate piecemeal necrosis	Severe focal cell damage
4	Severe piecemeal necrosis	Damage includes bridging necrosis
Stage	Fibrosis	
0	None	
1	Enlarged, fibrotic portal tracts	
2	Periportal or portal-portal septa but intact architecture	
3	Fibrosis with architecture distortion but no obvious cirrhosis	
4	Probable or define cirrhosis	

The degree of liver tissue damage was expressed by inflammation grade (grading, G) and fibrosis stage (staging, S). Grading was based on the degree of necrosis and inflammation, assessing the activity of the disease; staging was based on the degree of fibrosis and formation of cirrhosis, indicating the progress of the disease

Supplementary Table 4: Mean c and φ measurements of subgroups in fibrosis or inflammation.

Stage of fibrosis	c (m/s)	φ (rad)	Kruskal-Wallis Test for distinguishing with c			
			S(0-1)	S2	S3	S4
S0-1 (n = 35)	1.54 ±0.19	0.66±0.09	/	<0.05*	<0.05*	<0.05*
S2 (n = 37)	1.71±0.18	0.70±0.07	<0.05*	/	<0.05*	<0.05*
S3 (n = 18)	1.97±0.28	0.76±0.09	<0.05*	<0.05*	/	<0.05*
S4 (n = 80)	2.14±0.35	0.82±0.11	<0.05*	<0.05*	<0.05*	/
**Grade of inflammation**	**c (m/s)**	**φ (rad)**	**Kruskal-Wallis Test for distinguishing with φ**			
			G(0-1)	G2	G3	G4
G0-1 (n = 54)	1.75±0.29	0.70±0.08	/	<0.05*	<0.05*	<0.05*
G2 (n = 82)	1.88±0.33	0.74±0.09	<0.05*	/	<0.05*	<0.05*
G3 (n = 21)	2.14±0.21	0.85±0.10	<0.05*	<0.05*	/	0.11
G4 (n = 13)	2.37±0.59	0.94±0.10	<0.05*	<0.05*	0.11	/

Note: *Statistically significantly difference between various fibrosis and inflammation groups ($p < 0.05$)

c = shear wave speed, φ = loss angle of the complex shear modulus, S = staging, fibrosis stage, G = grading, inflammation grade

Supplementary Table 5: Univariable and Multivariate Logistic Regression Analyses of Variables Associated with fibrosis and inflammation in Training cohort

	Univariate		Multivariate			Univariate		Multivariate	
Parameter	Odds Ratio	*p*-Value	Odds Ratio	*p*-Value		Odds Ratio	*p*-Value	Odds Ratio	*P* Value
≥S1					≥G2				
c	13957.87	<0.05*	13957.87	<0.05*	*c*	8.92	<0.05*		
φ	45896.92	<0.05*			*φ*	2468.83	<0.05*	2468.83	<0.05*
≥S2					≥G3				
c	328.97	<0.05*	167.82	<0.05*	*c*	23.90	<0.05*		
φ	67534.71	<0.05*			*φ*	11525	<0.05*	11525	<0.05*
S3					G4				
c	632.50	<0.05*	376.04	<0.05*	*c*	17.57	<0.05*		
φ	544629.32	<0.05*			*φ*	10158	<0.05*	10158	<0.05*
S4									
c	137.42	<0.05*	70.67	<0.05*					
φ	144369.23	<0.05*							

Note: *Statistically significantly difference between fibrosis and inflammation groups ($p < 0.05$)

c = shear wave speed, *φ* = loss angle of the complex shear modulus, S = staging, fibrosis stage, G = grading, inflammation grade
